# Translational Medicine: Creating the Crucial Bidirectional Bridge between Bench and Bedside

**DOI:** 10.3390/ijms17111918

**Published:** 2016-11-16

**Authors:** Xiaofeng Jia

**Affiliations:** 1 Department of Neurosurgery, University of Maryland School of Medicine, Baltimore, MD 21201, USA; xjia@som.umaryland.edu; Tel.: +1-410-706-5026; 2 Department of Orthopaedics, University of Maryland School of Medicine, Baltimore, MD 21201, USA; 3 Department of Anatomy and Neurobiology, University of Maryland School of Medicine, Baltimore, MD 21201, USA; 4 Department of Biomedical Engineering, The Johns Hopkins University School of Medicine, Baltimore, MD 21205, USA; 5 Department of Anesthesiology and Critical Care Medicine, The Johns Hopkins University School of Medicine, Baltimore, MD 21205, USA

The life sciences are now entering a revolutionary era that calls for acceleration in understanding the complexity of biological systems and expands the need for more effective strategies for pursuing translational medicine [1]. The term Translational Medicine was introduced in the 1990s and became popular in the early 2000s. Translational research aims to reduce disease incidence, morbidity, and mortality by transforming scientific discoveries from laboratory, clinical, or population studies into novel strategies and clinical tools that improve human health [2]. Crossing the “valley of death”—the gap between excellent bench research and unsatisfied clinical need [3]—*Translational Medicine* aims to promote the transformation of basic science and clinical discoveries towards an ultimate goal: enhancing human health through advancing science.

Seeking to coordinate state of the art knowledge in basic science and clinical practice, *Translational Medicine* takes observations and challenges in clinical practice, and uses them to help form scientific hypotheses in the laboratory. Bench research investigators can then investigate and validate these hypotheses to develop new and effective clinical strategies. Therefore, *Translational Medicine* creates a crucial bidirectional link between bench and bedside: clinical insights are shared with researchers from bedside to bench to direct research topics, novel scientific discoveries and clinical strategies are provided for clinicians from bench to bedside to better human health, and after implementing new strategies, more insights are fed back to researchers from bedside to bench to further this process of advancing science and improving human health. The net result is a continuous and self-sustaining process that increases the efficiency of making clinically relevant discoveries in the laboratory that can readily benefit patient health in clinical practice.

Translational Medicine is a new and important field in medicine. It is highly interdisciplinary, aiming to ultimately improve human health by fostering multidirectional integration of basic laboratory research, patient-oriented clinical research, and population-based epidemiology research. The European Society for Translational Medicine states that “the goal of translational medicine is to combine disciplines, resources, expertise, and techniques within these pillars to promote enhancements in prevention, diagnosis, and therapies” [4]. Using novel interdisciplinary approaches, *Translational Medicine* includes but is not limit to: the development and clinical application of new biomarkers; the discovery, production, regulation and exploration of pharmacologic agents that improve clinical outcome; the discovery of new diagnostic tools and therapeutic interventions; the critical progress of computational approaches for genomics and bioinformatics; and the advancement of neuroengineering and new technologies in tissue engineering [5].

*Translational Medicine* is the new Section of the *International Journal of Molecular Sciences* (*IJMS*). It is being launched to cover critical steps of bridging bench and bedside to facilitate the characterization of disease model, support the generation of novel hypotheses, and help researchers more efficiently access and apply new findings across multiple disciplines. Published articles will include not only research findings from bench research on genes, cells, tissues, organs, and preclinical study in animals, but also new insights from clinical research on patients and populations. Covering a wide gamut of research findings will optimize and promote the bidirectional link between bench and bedside. This journal aims to create an effective platform that promotes all stages of interdisciplinary discovery, development, and validation of novel clinical strategies to serve basic science researchers, physician scientists, policy-makers, business developers, funding agencies, and most importantly, patients through advancing science. As the chief editor, I am confident that the excellent heritage of IJMS will be continued and advanced by this new section, and that *Translational Medicine* will be a highly valuable resource for both *IJMS* readers and authors.
